# Colonic diverticulitis location is a risk factor for recurrence: a multicenter, retrospective cohort study in Asian patients

**DOI:** 10.1038/s41598-022-08708-w

**Published:** 2022-03-16

**Authors:** Chih-Wei Sung, Kao-Lang Liu, Hsiu-Po Wang, I.-Chung Chen, Edward Pei-Chuan Huang, Wan-Ching Lien, Chien-Hua Huang

**Affiliations:** 1 Department of Emergency Medicine, National Taiwan University Hsin-Chu Hospital, Hsinchu, Taiwan; 2 Department of Medical Imaging, National Taiwan University Hospital, and National Taiwan University College of Medicine, Taipei, Taiwan; 3 Division of Gastroenterology and Hepatology, Department of Internal Medicine, National Taiwan University Hospital and National Taiwan University, Taipei, Taiwan; 4 Department of Emergency Medicine, National Taiwan University Hospital, Yun-Lin Branch, Taipei, Taiwan; 5 Department of Emergency Medicine, National Taiwan University Hospital and National Taiwan University, No.7, Chung-Shan South Road, Taipei, 100 Taiwan

**Keywords:** Health care, Medical research

## Abstract

Evidence regarding the recurrence of diverticulitis is limited in Asian patients. This study aims to investigate recurrence rates and identify predictive factors for the recurrence of diverticulitis following successful nonoperative treatment in Asian patients. A multicenter, retrospective cohort study was conducted between 2012 and 2018. Adult patients with computed tomography (CT)-proven colonic diverticulitis were included. The primary outcome was the recurrence of diverticulitis, which was defined as another episode of occurrence of the infection after index hospital stay. Cumulative recurrence rates were calculated using the Kaplan–Meier method. Cox regression models were employed to identify parameters that significantly and independently predicted recurrence. Hazard ratios (HRs) and 95% confidence intervals (CIs) were calculated. A total of 929 patients were included. Diverticulitis in the cecum/ascending occurred in 675 (72.6%) patients. The average follow-up period was 651 days. Recurrence was observed in 115 (12.4%) patients and most significantly observed in patients with sigmoid diverticulitis (HR, 2.24; 95% CIs 1.59–3.97), followed by those with descending colon diverticulitis (HR, 1.92; 95% CIs 1.17–3.25). Although most of the Asian patients had right-sided colonic diverticulitis, those with sigmoid diverticulitis had the highest risk of recurrence.

## Introduction

Acute colonic diverticulitis is an inflammatory disease characterized by microscopic and macroscopic perforations of the diverticular wall, with an occurrence of 10–25% in patients with diverticulosis^1^. Among those with diverticulitis, 15–20% develop severe complications, thereby contributing to mortality of approximately 5%^2^. However, even with a low mortality rate, diverticulitis can lead to various morbidities, including pelvic abscess, intestinal perforation, bowel fistula, bowel obstruction, peritonitis, and sepsis^3^.

Previous studies have reported diverse recurrence rates of diverticulitis, ranging from 1.6 to 60.5%^4–9^. Age and complicated diverticulitis with abscess formation were related to recurrence in western countries^5,7–9^. Furthermore, variation in the location of diverticulitis is another crucial factor suggesting that the inflammation reaction may differ between ethnicities^10^. In eastern countries, diverticulitis occurs more in the right-sided colon as compared with western countries^11^. Kim et al.reported that the recurrence of right colon diverticulitis was associated with smoking and the length of hospital stay during the first attack^12^. Even a recent large-scale ongoing prospective study (DAMASCUS) recruited Asians, the recurrence rate and predictive factors for recurrent diverticulitis as well as the relationship between the location and recurrence of diverticulitis have not been thoroughly investigated in Asian patients^13^.

We herein conducted a retrospective multicenter study to investigate the recurrence rate of diverticulitis following nonoperative treatments and possible predictive factors for recurrence in Asian patients.

## Methods

### Study design

This multicenter, retrospective cohort study was conducted at a medical center, and its two branches both providing tertiary medical care, from March 2012 to December 2018. This study was approved by the institutional review board of the National Taiwan University Hospital and waived the need for informed consent. All methods were performed in accordance with the relevant guidelines and regulations. It was also registered at ClinicalTrials.gov (NCT04500405). This work has been reported in line with the STROCSS criteria^14^.

### Study population

Adult, nonoperative patients more than 20 years of age with computed tomography (CT)-proven colon diverticulitis were eligible. Colonic diverticulitis was defined as colonic wall thickening (≥ 3 mm on the short axis of the lumen) and pericolic fat stranding identified through CT and confirmed by a certified radiologist who was not involved in patient enrollment and analysis. Patients who underwent nonoperative treatment including adequate hydration, nutrition, antibiotic treatment, and medication for pain control, were included. Pregnant patients, patients who aged less than 20 years or received colonic resection during the index hospital stay were excluded.

### Measurements

The included patients have completed the treatment and were cured during the index hospital stay. The total follow-up duration was defined as the number of days starting from one month after hospital discharge to recurrence, or the end date of the study (December 31, 2018). The patients were free of diverticulitis when follow-up started. The recurrence of diverticulitis was defined as the presence of recurrent clinical symptoms and CT-verified diverticulitis one month after the index hospital stay. The recurrent site was confirmed based on the certified radiologist’s formal CT report. In case of any inconsistency in the location of diverticulitis, two radiologists would discuss and make the final decision.

The clinical data were obtained from electronic medical records: age, sex, body mass index (BMI), comorbidities, presenting symptoms (fever, abdominal pain, abdominal fullness, nausea/vomiting, anorexia, dysuria, urinary frequency, constipation, and diarrhea), laboratory data (white blood cells and its band form, C-reactive protein, creatinine, and amylase), diverticulitis types (including locations and complications), total antibiotic treatment duration, length of in-hospital stay, follow-up duration, and the status and time of recurrence. Complications included phlegmon or abscess followed by peritonitis, obstruction, and fistula. A research assistant who was not responsible for statistical analysis recorded the variables in a predefined datasheet.

### Outcomes

The primary outcome was the recurrence of diverticulitis. The secondary outcomes were possible predictors of recurrence.

### Statistical analysis

All data were analyzed using SAS software (SAS 9.4, Cary, North Carolina, USA). Categorical variables and continuous variables were presented as numbers (percentages) and mean ± standard deviation, respectively. The Chi-square test was used for analyzing categorical variables. Kruskal–Wallis analysis was performed to test the normality of the variables. When the continuous variables showed normal distribution, a student *t*-test was used. Otherwise, the Mann–Whitney U test was used to compare outcomes between two independent groups.

The follow-up data were censored when recurrence or at the end date of the study (December 31, 2018). The cumulative recurrence rates were calculated using the Kaplan–Meier method. The Cox regression models were employed to identify parameters that significantly and independently predict recurrence. Covariates in the model were age, sex, BMI, comorbidities, presenting symptoms, laboratory data, diverticulitis types, total antibiotic treatment duration, and hospitalization duration. The hazard ratio (HR) and 95% confidence intervals (CIs) were computed. The cumulative recurrence rates were calculated for the significant predictive factor and the recurrence-free curves were displayed and compared using the log-rank test. A p-value of less than 0.05 was considered statistically significant.

### Consent for publication

All authors approve this version for publication and are accountable for its content.

## Results

### Patient flow and baseline characteristics

**Figure 1:**
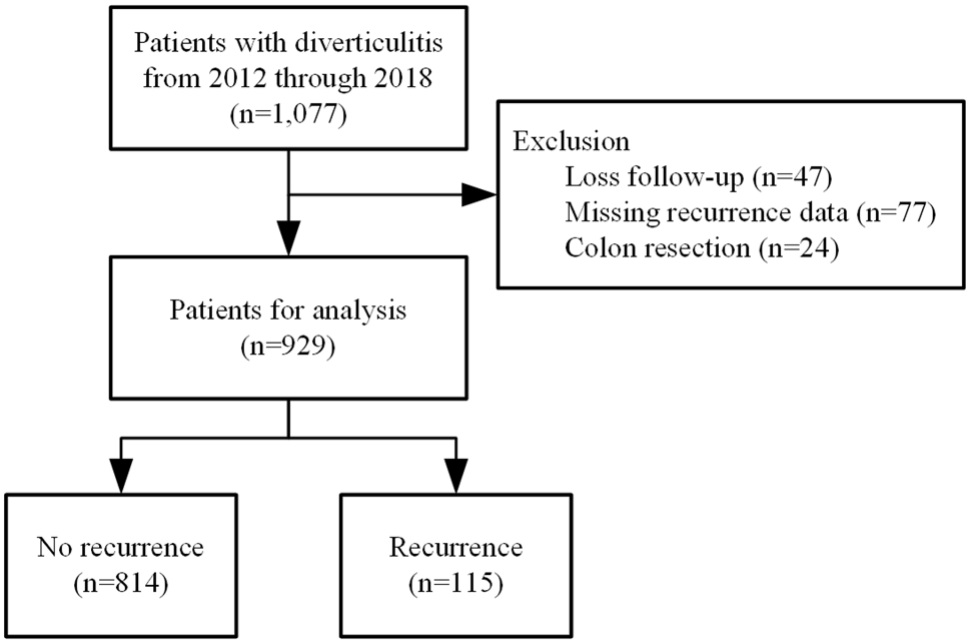
The study flowchart.

A total of 1077 patients had diverticulitis during the study period, and they were free of diverticulitis when follow-up started. Among them, 148 patients were excluded because of loss of follow-up (n = 47), missing data (n = 77), and receiving colon resection (n = 24, Supplementary Table). Finally, 929 patients who completed the nonoperative treatment were included in the analysis. One hundred and fifteen patients (12%) suffered recurrent diverticulitis (Fig. 1).

The mean age was 52.9 ± 16.3 years, and 52% of them were males. The average BMI was 24.3 ± 3.8 kg/m^2^. Over 70% of the patients had diverticulitis in the cecum/ascending colon. Complicated diverticulitis occurred in 130 patients (14.0%).

After preliminary examination of normality in each variable, the characteristics were compared between patients with and without recurrence (Table 1). Because acute diverticulitis was relatively rare in patients younger than the age of 40^15^, we stratified the patients into 3 age categories: 20–39 (younger group), 40–64 (middle age group), and more than 65 years (older group). Older patients and patients with hypertension, diabetes, coronary artery disease/cerebrovascular events, and longer antibiotic treatment had more possibility of recurrence as compared with those in the non-recurrence group. Although more than 70% of diverticulitis occurred at the cecum/ascending colon, the sigmoid and descending colon diverticulitis had a higher chance of recurrence.

**Table 1:** Demographics of the included patients.

Variables		Recurrence		
	Total	No	Yes	*p*-value^‡^
	(n = 929)	(n = 814)	(n = 115)	
**Age, years**				< 0.01
20–39	203 (22%)	185 (23%)	18 (16%)	
40–64	438 (47%)	390 (48%)	48 (42%)	
≧65	288 (31%)	239 (29%)	49 (43%)	
Male gender, n (%)	484 (52%)	424 (52%)	60 (52%)	0.75
Body mass index, kg/m^2^*	24.3 ± 3.8	24.2 ± 4.8	24.6 ± 3.6	0.71
**Comorbidities, n (%)**				
Diabetes mellitus	102 (11%)	78 (10%)	24 (21%)	< 0.01
Hypertension	272 (29%)	223 (27%)	49 (43%)	< 0.01
CAD or CVA^†^	76 (8%)	57 (7%)	19 (17%)	< 0.01
Liver cirrhosis	15 (2%)	14 (2%)	1 (0.9%)	0.56
**Symptoms, n (%)**				
Abdominal pain	719 (77.%)	644 (79%)	75 (65%)	0.65
Abdominal fullness	23 (3%)	18 (2%)	5 (4%)	0.31
Nausea/vomiting	116 (13%)	101 (12%)	15 (13%)	0.59
Anorexia	70 (8%)	68 (8%)	2 (2%)	0.18
Dysuria	29 (3%)	25 (3%)	4 (3%)	0.08
Urinary frequency	19 (2%)	15 (2%)	4 (3%)	0.50
Constipation	28 (3%)	26 (3%)	2 (2%)	0.68
Diarrhea	106 (11%)	91 (11%)	15 (13%)	0.78
Fever	186 (20%)	166 (20%)	20 (17%)	0.12
Laboratory data*				
White blood cells, × 10^3^/uL	12.6 ± 7.4	12.9 ± 7.2	11.9 ± 3.2	0.08
Band, %	0.3 ± 3.7	0.2 ± 2.9	0.4 ± 2.2	0.27
CRP^†^, mg/dL	6.9 ± 6.8	7.9 ± 5.9	4.9 ± 5.7	0.11
Creatinine, mg/dL	1.0 ± 1.2	1.1 ± 1.2	0.9 ± 0.5	0.28
Amylase, U/L	43.9 ± 19.8	46.2 ± 12.4	34.2 ± 12.4	0.10
Complicated diverticulitis, n (%)	130 (14%)	112 (14%)	18 (16%)	0.17
**Location, n (%)**				< 0.01
Cecum/ascending colon	675 (73%)	628 (77%)	47 (41%)	
Transverse colon	29 (3%)	27 (3%)	2 (2%)	
Descending colon	88 (10%)	67 (8%)	21 (18%)	
Sigmoid colon	137 (15%)	92 (11%)	45 (39%)	
Antibiotics duration, days*	10.2 ± 8.9	7.0 ± 8.8	12.9 ± 11.4	0.01
Hospitalization, days*	7.1 ± 8.2	6.2 ± 7.5	11.2 ± 13.2	< 0.01
Follow-up, days*	651.7 ± 617.0	653.1 ± 607.5	648.2 ± 552.6	0.75

*Presented with mean ± standard deviation.

^†^CAD, coronary artery disease; CVA, cerebrovascular accident; CRP, C-reactive protein.

^‡^Comparisons between the recurrence and the non-recurrence groups.

### Recurrence of diverticulitis

The cumulative recurrence rates of potential risk factors and the association between the factors and recurrence were demonstrated in Table 2. In univariate analysis, potential factors such as age, diabetes mellitus, hypertension, ischemic diseases, antibiotic use, hospitalization, and diverticulitis location were associated with the recurrence of diverticulitis.

**Table 2:** Risk factors for recurrence of colonic diverticulitis.

Variables	Cumulative recurrence rates	Univariate analysis		Multivariate analysis	
		Hazard ratio (95% CIs)	*p-*value	Hazard ratio (95% CIs)	*p-*value
Age, years*					
20–39	21%	Reference	-	Reference	-
40–64	28%	1.76 (1.09–2.08)	0.02	1.42 (1.18–1.68)	0.02
≧65	31%	2.85 (1.71–3.17)	< 0.01	2.27 (1.98–2.59)	< 0.01
Diabetes mellitus	29%	1.87 (1.23–2.45)	< 0.01	1.78 (0.99–2.96)	0.06
Hypertension	11%	1.28 (1.03–2.75)	0.03	0.85 (0.53–1.39)	0.41
CAD or CVA^†^	8%	2.09 (1.21–3.43)	< 0.01	1.65 (0.88–3.01)	0.11
Antibiotics duration	-	0.82 (0.76–0.99)	0.01	0.98 (0.96–1.03)	0.35
Hospitalization	-	1.21 (1.03–1.69)	0.02	1.04 (0.98–1.07)	0.06
Location*					
Cecum/Ascending	8%	Reference	-	Reference	-
Transverse	12%	0.87 (0.08–4.23)	0.79	0.72 (0.07–4.88)	0.81
Descending	46%	2.32 (1.21–3.61)	< 0.01	1.92 (1.17–3.25)	0.01
Sigmoid	68%	2.96 (1.48–4.27)	< 0.01	2.24 (1.59–3.97)	< 0.01

*Remained significant after adjusting other confounders.

^†^CAD, coronary artery disease; CVA, cerebrovascular accident.

Age and the location of index diverticulitis were associated with the recurrence after adjusting the other confounders. As compared with the younger group, patients in the older age group had a significantly higher chance for recurrence (HR, 2.27, 95% CIs, 1.98–2.59, *p* < 0.01). Patients in the middle-age group also had a significant impact on recurrent diverticulitis (HR, 1.42, 95% CIs, 1.18–1.68, *p* = 0.02). As compared with diverticulitis in the ascending colon, the rate of recurrence was significantly higher in patients who had diverticulitis in the descending or sigmoid colon (HR, 1.92, 95% CIs, 1.17–3.25, p = 0.01 in descending colon; HR, 2.24, 95% CIs, 1.59–3.97, p < 0.01 in sigmoid colon).

**Figure 2:**
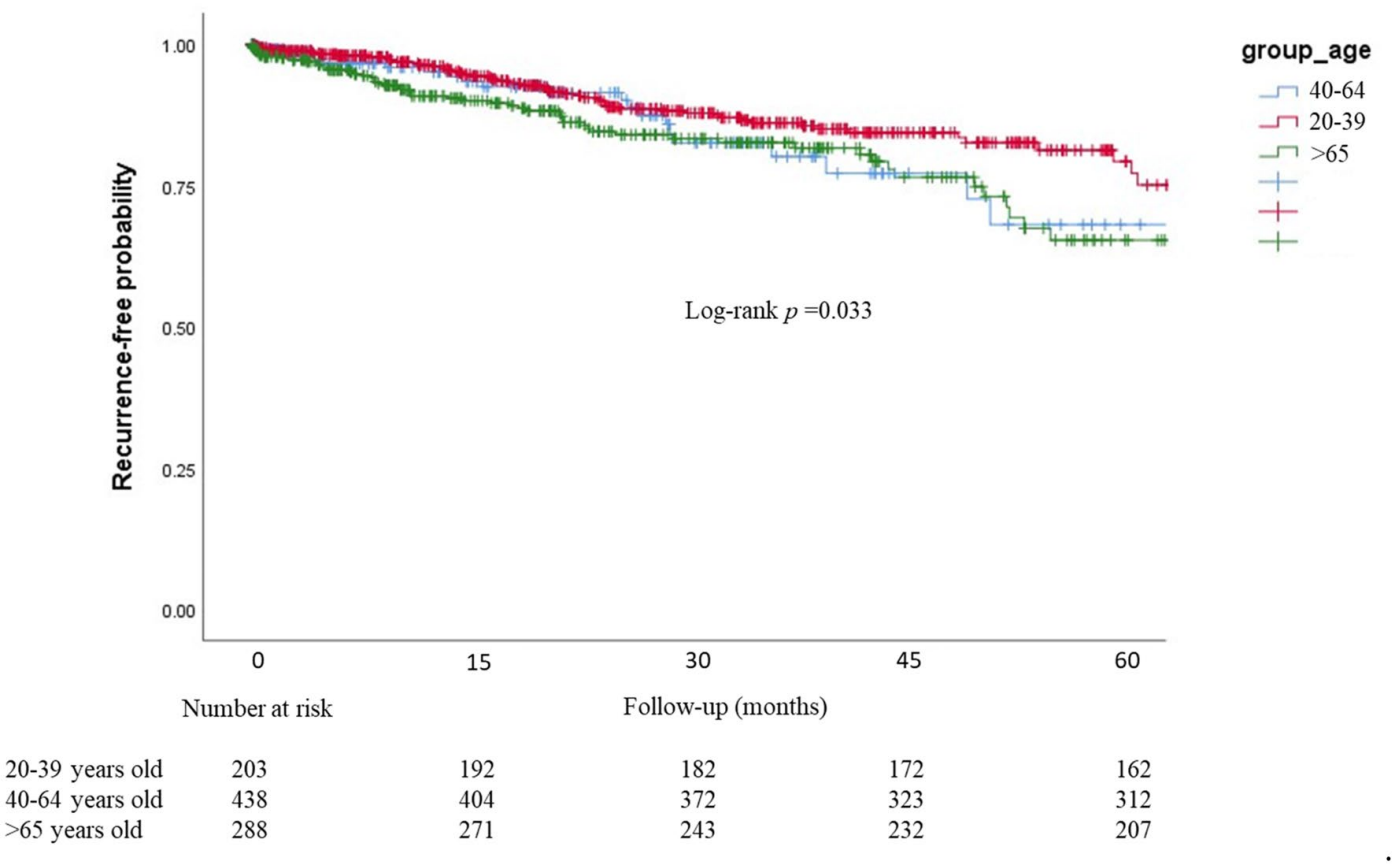
The Kaplan–Meier plot for recurrence free probability stratified by age groups during follow-up.

**Figure 3:**
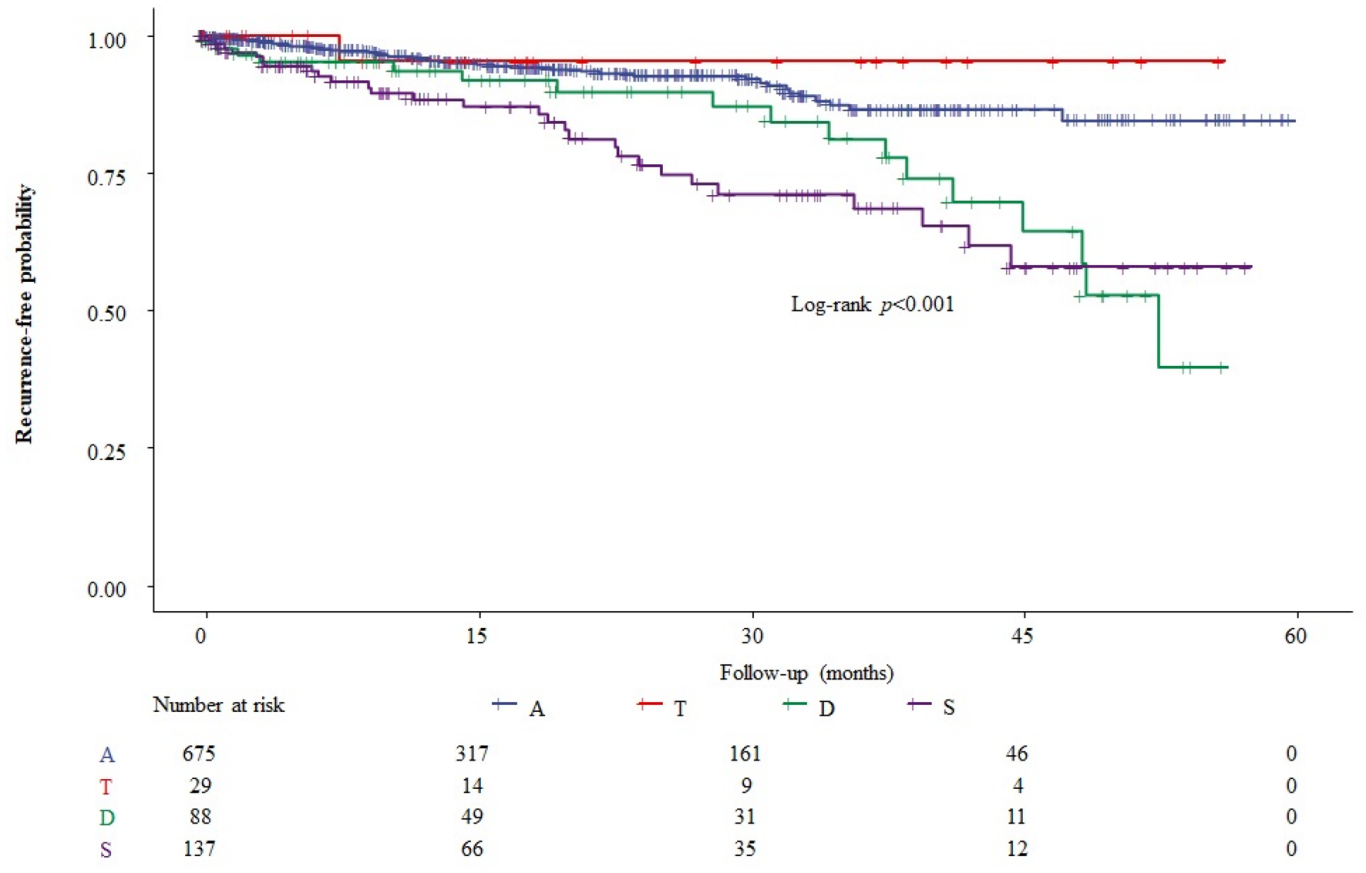
The Kaplan–Meier plot for recurrence free probability stratified by location of diverticulitis during follow-up.

Figures 2 and 3 illustrates the recurrence-free probability stratified by the age group and location, respectively. In Fig. 2, we found that the older group had a higher probability of recurrence, compared with the younger group (log-rank test, p = 0.033). Patients with diverticulitis located in the descending or sigmoid colon exhibited a significantly higher recurrence rate than those with diverticulitis in the ascending colon (Fig. 3, log-rank test, *p* < 0.001).

The cumulative recurrence rates at different age groups and locations at different time points were listed in Table 3. Generally, patients with sigmoid colon diverticulitis had higher recurrence rate. One-fifth of patients with initial colon diverticulitis experienced recurrence in 2 years.

**Table 3:** The recurrence rates for patients with different locations and age categories.

	6-month	12-month	24-month
**Locations**			
Cecum/ascending colon	4%	2%	3%
Transverse colon	0	8%	5%
Descending colon	18%	14%	13%
Sigmoid colon	20%	20%	21%
**Age groups (years old)**			
20–39	3%	5%	8%
40–64	5%	7%	11%
≧65	3%	4%	13%

### Patients with colon resection

Among these 24 patients receiving colon resection during the index hospital stay, 10 (41.7%) patients had complicated diverticulitis (Supplementary Table 1). The majority of the diverticulitis occurred over the sigmoid colon (58%) and descending colon (25%). The average duration of antibiotic treatment was 11 days.

## Discussion

This study investigates the outcomes of nonoperative management in adult patients with acute colonic diverticulitis. Over 70% of the diverticulitis occurred over the right-sided colon. Fourteen percent of the patients had complicated diverticulitis. The overall recurrent rate was 12%. Older patients and patients with sigmoid or descending colon diverticulitis experienced a higher chance of recurrence.

In the current study, the location of diverticulitis was an independent risk factor for its recurrence among Asian patients. Although acute diverticulitis was more common on the right-sided colon, this location presented a relatively minor risk for recurrence and favorable outcomes^16^. In contrast, patients with diverticulitis in the sigmoid colon and descending colon had a higher risk of recurrence. To the best of our knowledge, this study was the first to demonstrate the location of diverticulitis having an impact on the recurrence of diverticulitis in Asian patients.

Stratified by the location, the cumulative recurrent rates in right-sided diverticulitis was 8%, 46% in the descending colon, and 68% in the sigmoid colon (Tale 2). As compared with the meta-analysis conducted by Lee et al., the recurrence rate of right-sided diverticulitis was relatively lower (12% in the meta-analysis)^17^. One of the possible reasons is ethnicity. Previous studies as well as our results showed that diverticulitis over the ascending colon was more common in Asian countries than in western countries^12,18–20^. However, risk factors of recurrence would differ between right- and left-sided colon diverticulitis. The risk factors for the recurrence of right-sided diverticulitis included smoking, hospital stay duration during the first admission, multiple diverticula, and intraperitoneally located diverticulitis^12,20^. In contrast, young age and abscess formation were top risk factors for the recurrence of left-sided diverticulitis^9^. After adjusting the confounders in this study, the location remained a significant factor for recurrence among Asian patients.

Another reason may be the complexity of the disease. Previous studies showed that patients with complicated diverticulitis had a higher risk of recurrence, especially those with left-sided diverticulitis^2,20–22^. Abscess formation, one of the most common complications, causes inflammatory extension at index episode, which further increases the treatment course and delays discharge from the hospital. The incidence of complicated diverticulitis varied as per country and ethnicity^23^. Ho et al. reported that patients with complicated diverticulitis at the first admission, especially those with abscesses, have a high risk of recurrence and poor outcomes and should be offered surgery^7^. In the present study, there was no significant relation between complicated diverticulitis and recurrence, which can be explained by the lower rate of complicated diverticulitis and the location of diverticulitis. Complicated diverticulitis occurred mostly in the left-sided colon^24^.

Published data regarding the effect of age on the risk of recurrent diverticulitis were controversial.Hupfeld et al. reported there was a high likelihood of young age associated with recurrent diverticulitis^9^. Van Dijk et al. reviewed 4 studies using survival analyses and demonstrated that age was not a risk factor for recurrence^25^.Notably, most of the studies included the western population. Colonic diverticulitis may have different clinical features in different ethnicities. Diverticulitis in young Asians is often right-sided, mild in severity, and recurrent attacks are uncommon^26^. In our study, older patients had a higher chance of recurrent diverticulitis.

Some limitations exist in this study. First, this is a retrospective cohort study. Some important data could not be gathered such as the daily habits of the patients (i.e. smoking), thus failing to determine whether smoking is an independent factor for recurrence in Asian patients^12^. Furthermore, we only recorded the main location of diverticulitis. The number of the diverticulum was not recorded. Second, selection bias may exist. Our results may not reflect the epidemiological profile of the entire population of the whole country. Our hospital and its affiliated hospitals were academic hospitals providing tertiary health care where the disease pattern would be more severe and complicated. Therefore, the recurrence rate might be overestimated in this study. Third, we could not evaluate patients who had recurrence at other hospitals. This might underestimate the recurrence rate and risk effects. Last, missing data, such as diet conditions and family history, is another cause of concern.

In conclusion, over 70% of diverticulitis occurred in the ascending colon. However, patients with sigmoid or descending colon diverticulitis have a higher risk of recurrence. Older patients had higher recurrence rate. Further prospective multicenter studies aimed at assessing the risk factors of recurrence of secondary diverticulitis are required to overcome the limitations of our study.

## Supplementary Information

Supplementary Information.

## Data Availability

The data underlying this article will be shared on reasonable request to the corresponding author.

## Abbreviations

BMI: Body mass index
CI: Confidence intervals
CT: Computed tomography
HR: Hazard ratios
