# Author Correction: Colonic diverticulitis location is a risk factor for recurrence: a multicenter, retrospective cohort study in Asian patients

**DOI:** 10.1038/s41598-022-17571-8

**Published:** 2022-08-02

**Authors:** Chih-Wei Sung, Kao-Lang Liu, Hsiu-Po Wang, I.-Chung Chen, Edward Pei-Chuan Huang, Wan-Ching Lien, Chien-Hua Huang

**Affiliations:** 1 Department of Emergency Medicine, National Taiwan University Hsin-Chu Hospital, Hsinchu, Taiwan; 2 Department of Medical Imaging, National Taiwan University Hospital, and National Taiwan University College of Medicine, Taipei, Taiwan; 3 Division of Gastroenterology and Hepatology, Department of Internal Medicine, National Taiwan University Hospital and National Taiwan University, Taipei, Taiwan; 4 Department of Emergency Medicine, National Taiwan University Hospital, Yun-Lin Branch, Taipei, Taiwan; 5 Department of Emergency Medicine, National Taiwan University Hospital and National Taiwan University, No.7, Chung-Shan South Road, Taipei, 100 Taiwan

Correction to: *Scientific Reports* 10.1038/s41598-022-08708-w, published online 16 March 2022

The original version of this Article contained an error in Reference 6, which was incorrectly given as

Gill, H. *et al.* Sedation management during therapeutic hypothermia for neonatal encephalopathy: Atropine premedication for endotracheal intubation causes a prolonged increase in heart rate. *Resuscitation* **85**, 1394–1398 (2014).

The correct reference is listed below:

Park, S. J., Choi, S. I., Lee, S. H., & Lee, K. Y. Image-guided conservative management of right colonic diverticulitis. *World J Gastroenterol.* **15**, 5838–5842 (2009).

Additionally, the Funding section was omitted. The Funding section now reads:

“Financial or grant support: The Ministry of Science and Technology, Taiwan (MOST 110-2511-H-002-009-MY2).”

The original Article has been corrected.

