# Supplementary material for: Colonic diverticulitis location is a risk factor for recurrence: a multicenter, retrospective cohort study in Asian patients
Source: Sci Rep. 2022 Mar 16;12:4559. doi: 10.1038/s41598-022-08708-w (PMC8927129; doi:10.1038/s41598-022-08708-w)
Supplement: Supplementary file 1 — Supplementary Information. [file 41598_2022_8708_MOESM1_ESM.docx]

| Supplementary Table 1. Characteristics of patients undergoing colon resection | |
| --- | --- |
| Variables | Patients with resection |
|  | (n= 24) |
| Age, years^*^ | 52.6±17.2 |
| Male gender, n (%) | 11 (46%) |
| Body mass index, kg/m^2*^ | 24.4±4.1 |
| Fever, n (%) | 5 (21%) |
| White blood cells, x10^3^/uL^*^ | 12.5±5.2 |
| Band, %^*^ | 1.4±4.0 |
| CRP^*†^, mg/dL | 6.4±5.4 |
| Complicated diverticulitis, n (%) | 10 (42%) |
| Location, n (%) |  |
| Cecum/Ascending colon | 3 (13%) |
| Transverse colon | 1 (4%) |
| Descending colon | 6 (25%) |
| Sigmoid colon | 14 (58%) |
| Antibiotics duration, days^*^ | 11.0±9.4 |
| Hospitalization, days ^*^ | 8.4±10.2 |
| Diverticulitis occurred over the preserved colon during follow-up, % | 0 |
| ^*^Presented with mean ± standard deviation.  ^†^CRP, C-reactive protein. | |
